# Flowers and Paintings

**Published:** 1873-01

**Authors:** 


### FLOWERS AND PAINTINGS

INDICATE taste, elevation and culture. If there is ever occasion to stop at a "Country Inn," when traveling, give preference to the house which shows a flower in the window, or about the door, for there is cleanliness within, a snowy table cloth and a well spread bed.

When Mungo Park seemed at the very point of death from exhaustion and hopelessness, in the midst of African desolation, he spied, coming up from the sand, the tiniest flower, but it was most beautiful; the thought instantly came to him, "If the Omnipotent One takes care of this lonely flower in such a place as this, surely He will care for me, His creature and His child." In an instant, as with an inspiration, he was nerved to hope, and within an hour help came. Flowers carry us back to childhood, for it was then their freshness and their beautiful bright tints made their ineffaceable impressions of sweetness and purity, and led the mind reverently upward, in contemplation of the skill which formed them and of the benevolence which clothed them in such heavenly hues. If possible, then, let a growing flower be always in sight, and whenever practicable have a bouquet or a single pink or rose on the table, morning, noon and night; it will give an air of cheeriness, of animation, of purity and of good will, which will richly pay the cost of time and trouble in plucking it from the stem in the garden or conservatory.

One of the most pleasant of friendship's reminders is sending a fresh flower to the sick chamber of those we love; any where and every where it is a good letter of introduction.

It was the uniform custom of Lord Bacon to have fresh flowers on his table, morning, noon and night. Leigh Hunt well said, "Set flowers on your table, a whole nosegay if you can get it; or but two or three, or a single flower, a rose, a pink, a daisy or a buttercup, and keep them alive in a little water; aye, preserve a bunch of clover or a handful of flowering grass, one of the most elegant of nature's productions, and you have something on your table which reminds you of God's creation and gives you a link with the poets who have done it most honor."

